# Malignant Catatonia Versus Neuroleptic Malignant Syndrome

**DOI:** 10.7759/cureus.15818

**Published:** 2021-06-21

**Authors:** Saral Desai, Tinu Hirachan, Anca Toma, Adela Gerolemou

**Affiliations:** 1 Psychiatry, Brookdale University Hospital Medical Center, Brooklyn, USA

**Keywords:** neuroleptic malignant syndrome (nms), subtypes of catatonia, clinical case report, atypical antipsychotic, schizophrenia and other psychotic disorders, adverse event, diferential diagnosis, dantrolene, consultation liaison psychiatry, emergency psychiatry

## Abstract

The clinical presentations of neuroleptic malignant syndrome (NMS) and malignant catatonia (MC) are similar, posing a diagnostic challenge. Here, we present a 58-year-old Caucasian male who presented to the emergency department with an altered mental state, fever, tachycardia, and rigidity. Labs were remarkable for elevated creatine phosphokinase (CPK) and leukocytosis. The patient was on a regimen of clozapine and cariprazine to manage schizophrenia, lorazepam to treat catatonia, and mirtazapine to treat insomnia and appetite stimulation. The patient was initially diagnosed with NMS after common metabolic, infectious, and substance-induced etiologies were ruled out. Within 72 hours of receiving dantrolene and lorazepam, the patient's fever, tachycardia, and other laboratory abnormalities resolved. However, when the patient's rigidity, waxy flexibility, mutism, and stupor persisted, the diagnosis was reconsidered and changed to MC. Our case discusses the overlapping clinical presentations of NMS and MC, demonstrating a diagnostic challenge.

## Introduction

Neuroleptic malignant syndrome (NMS) is characterized by distinctive clinical features such as altered mental status, muscular rigidity, hyperpyrexia, and dysautonomia [1]. NMS is, as the name implies, correlated to the use of neuroleptic medications [1]. NMS has been reported in 0.2% to 3% of patients taking antipsychotic medications [1]. Catatonia is most commonly observed in patients with schizophrenia, presenting with both mood and motor disturbance [2]. Malignant catatonia (MC) is a subtype of catatonia characterized by stupor, mutism, catalepsy, waxy flexibility, negativism, posturing, pyrexia, autonomic dysfunction, rigidity, and increased CPK levels [2-4]. Since NMS and MC can present similarly, diagnostic difficulties arise. We discuss an example of such a case below.

## Case presentation

A 58-year-old Caucasian male with a medical history of benign prostatic hyperplasia, hypertension, and psychiatric history of schizophrenia was brought to the emergency department (ED) with altered mental status. The nursing home staff called emergency medical services (EMS) after finding the patient lying on the floor in a confused state. According to the EMS report, the patient was completely mute, rigid, and tremulous.

The patient presented to the emergency department (ED) with a fever of 101 °F, tachycardia (123 bpm), tremors, and mutism. Physical exam demonstrated lead-pipe rigidity. Initial workup was significant for elevated white blood cell (WBC) count (18,500/mm3), neutrophil count (91.1 percent), and creatine phosphokinase (CPK) levels (1088 Units/L). Infectious, metabolic, and substance abuse-related causes were ruled out. Initial computed tomography (CT) scan of the head was limited due to motion artifacts. At this time, the neurology consult team suspected NMS. After discontinuing home medications, the patient was started on dantrolene 25 mg QD and lorazepam 2 mg QD for NMS. Although bromocriptine administration was indicated, nasogastric tube insertion was limited due to significant muscular rigidity.

On day three of hospitalization, CPK levels trended down to 211 Units/L, and his fever resolved. Dantrolene was discontinued while lorazepam 2 mg TID was continued. The patient's rigidity, mutism, stupor, and posturing persisted, and the patient’s diagnosis was reconsidered. Due to the patient's persistent catatonia without known recent changes in his home neuroleptic medications, we suspected the patient’s initial presentation was more consistent with a diagnosis of MC rather than NMS. Lorazepam was increased to 9 mg QD as indicated for refractory catatonia. As the patient's clinical condition gradually improved, his home schizophrenia medications were restarted - clozapine 25 mg QD and mirtazapine 7.5 mg for appetite stimulation and sleep. Lorazepam was discontinued due to adverse interaction with clozapine, and the dose of clozapine was increased to 50 mg. Following this regimen change, the patient once again became febrile, developed leukocytosis and worsening tachycardia. All psychiatric medications were discontinued, and a repeat head CT revealed no acute findings. These clinical findings resemble the patient’s initial ED presentation.

Collateral information was obtained from various sources, including nursing home staff, current caseworkers, and prior hospitalizations. Two months before presentation, the patient was transferred from a state psychiatric hospital to a long-term care facility. The patient was on a daily home regimen of clozapine 150 mg, fluphenazine 5 mg, and lorazepam 8 mg during his two-year stay in a state psychiatric institution. Fluphenazine was terminated when the patient was transferred to the nursing home. Clozapine was titrated down to 100 mg, while mirtazapine 7.5 mg and cariprazine 3 mg were initiated. For nearly two months before presentation, the patient was on a consistent dose of clozapine 100 mg, cariprazine 3 mg, mirtazapine 7.5 mg, and lorazepam 8 mg.

## Discussion

The pathophysiology of NMS remains unclear. Currently, the most accepted theory is the antagonism of D2 dopamine receptors [5,6]. It is hypothesized that inhibiting D2 receptors in the hypothalamus leads to temperature dysregulation with subsequent hyperpyrexia [5,6]. The inhibition of D2 receptors in the striatum results in muscular rigidity, while the inhibition of D2 receptors in the mesolimbic and cortical pathways results in altered mental status [5,6]. NMS is most frequently correlated with the use of potent first-generation antipsychotics, which block D2 receptors. However, it may occur with abrupt discontinuation of dopaminergic medications such as levodopa or amantadine [5,6]. Furthermore, NMS may also occur with second-generation antipsychotics, which have a lower affinity for the D2 receptor than first-generation neuroleptics and antidepressants, such as amoxapine, citalopram, and phenelzine [5,6]. 

According to the DSM-5, catatonia is diagnosed when three or more of the following symptoms are present: mutism, stupor, catalepsy, waxy flexibility, negativism, posturing, mannerism, stereotypy, unresponsive agitation, grimacing, echolalia, and echopraxia [7]. A diagnosis of malignant catatonia is made when a catatonic patient exhibits symptoms of autonomic instability such as fever, tachycardia, hypertension, diaphoresis, elevated CPK, leukocytosis, and low serum iron [8]. 

At first glance, NMS and MC are clinically similar with minor distinctions (Table 1). While autonomic instability may help differentiate NMS from non-malignant catatonia, discerning NMS from malignant catatonia requires recent exposure to dopamine blocking agents or sudden dopamine agonist withdrawal. Lead-pipe rigidity is more suggestive of NMS than MC [9].

**Table 1:** Differences and similarities between NMS and MC

NMS	MC
Elevated CPK	Elevated CPK
Lead-pipe rigidity, tremors	Waxy rigidity, tremors
Altered mental status	Altered mental status
Fever	Fever
Tachycardia	Tachycardia
Diaphoresis	Diaphoresis
Requires presences of neuroleptic medication within the past 72 hours, increased dosage of neuroleptics, or sudden withdrawal of dopaminergic agents.	Does not require the presence of a neuroleptic drug.

In this case study, the patient’s initial presentation was consistent with both NMS and MC. The presence of lead-pipe rigidity may have swayed the initial diagnosis toward NMS (Table 2). However, it is unusual for a patient to present with NMS after being on a consistent dose of clozapine, cariprazine, and mirtazapine for two months, as symptoms typically develop within weeks. Although second-generation antipsychotics have been associated with NMS, patients rarely develop NMS after two months. Clozapine is often associated with atypical NMS [10]. Secondly, the patient was on a steady dose of lorazepam 8 mg for two months, which is the mainstay treatment of catatonia. As such, the probability of MC on initial presentation is questioned, though not excluded. Alternatively, the patient may have developed NMS superimposed on catatonia, as individuals with catatonia are at greater risk of developing NMS [11].

**Table 2:** Signs and symptoms in our case present on ED admission

Signs & Symptoms	Condition
Fever (101 F)	Present in both conditions
Tremulousness	Present in both conditions
Tachycardia (123 bpm)	Present in both conditions
Altered mental status	Present in both conditions
Lead-pipe rigidity	Favors NMS
Elevated CPK (1088 U/L)	Present in both condition

Once the patient’s initial NMS resolved after administration of dantrolene, it is possible that underlying catatonia persisted. Another possibility that cannot be excluded is improper medication administration, such as missed doses of lorazepam 8 mg at the nursing home, which may have precipitated a relapse of MC [12]. It is also possible that lorazepam withdrawal in the presence of clozapine may have triggered episodes of either NMS or MC. Prior case reports suggest lorazepam may improve outcomes in both NMS and MC [13]. Ultimately, differentiating NMS from MC is challenging. Further research is needed to differentiate these two conditions and determine appropriate treatments.

## Conclusions

Malignant catatonia and neuroleptic malignant syndrome are two distinct conditions with a close overlap in the initial presentation. This poses diagnostic challenges for clinicians, especially in patients with a prior history of catatonia. Both of these life-threatening conditions require prompt diagnosis and treatment for improved prognosis, requiring more research to differentiate these conditions and uncover appropriate diagnostic strategies.
